# Lifestyle Factors in Hypertension Drug Research: Systematic Analysis of Articles in a Leading Cochrane Report

**DOI:** 10.1155/2014/835716

**Published:** 2014-01-28

**Authors:** Dan E. Wilson, Tashina Van Vlack, Brendin P. Schievink, Eric B. Doak, Jason S. Shane, Elizabeth Dean

**Affiliations:** ^1^Allan McGavin Sports Medicine Centre, University of British Columbia, Vancouver, BC, Canada V6T 1Z3; ^2^Father David Bauer Clinic (Centric Health Corporation), Calgary, AB, Canada T2N 3Y9; ^3^CBI Health Centre, Duncan, BC, Canada V9L 1N8; ^4^Squamish Physio & Wellness Centre, Squamish, BC, Canada V8B 0B4; ^5^Movéo Sport and Rehabilitation Centre, North Vancouver, BC, Canada V7L 2P7; ^6^Department of Physical Therapy, Faculty of Medicine, University of BC, Vancouver, BC, Canada V6T 1Z3

## Abstract

Established standards for first-line hypertension management include lifestyle modification and behavior change. The degree to which and how lifestyle modification is systematically integrated into studies of first-line drug management for hypertension is of methodological and clinical relevance. This study systematically reviewed the methodology of articles from a recent Cochrane review that had been designed to inform first-line medical treatment of hypertension and was representative of high quality established clinical trials in the field. Source articles (*n* = 34) were systematically reviewed for lifestyle interventions including smoking cessation, diet, weight loss, physical activity and exercise, stress reduction, and moderate alcohol consumption. 54% of articles did not mention lifestyle modification; 46% contained nonspecific descriptions of interventions. We contend that hypertension management research trials (including drug studies) need to elucidate the benefits and risks of drug-lifestyle interaction, to support the priority of lifestyle modification, and that lifestyle modification, rather than drugs, is seen by patients and the public as a priority for health professionals. The inclusion of lifestyle modification strategies in research designs for hypertension drug trials could enhance current research, from trial efficacy to clinical outcome effectiveness, and align hypertension best practices of a range of health professionals with evidence-based knowledge translation.

## 1. Introduction

Large-scale randomized controlled clinical trials (RCCTs) have long been considered the gold standard to evaluate treatment approaches to control blood pressure. Two streams of research have emerged: pharmacological and behavioral. Pharmacological research has generally investigated five classes of pharmaceuticals for blood pressure control: ACE inhibitors, angiotensin II receptor blockers, beta-receptor blockers, calcium channel blockers, and thiazide diuretics [1, 2]. Behavioral and lifestyle research has included smoking cessation, diet (including salt restriction), weight loss, physical activity and exercise, and decreased alcohol consumption. Both research streams have elucidated effective hypertension management and combined pharmaceuticals and behavioral modification may lead to superior outcome. RCCTs and systematic reviews based on such trials are considered the highest level of evidence for hypertension management.

Although there are several established first-line practice guidelines for the detection and management of hypertension, lifestyle behavior change is considered first line across levels of disease severity and concurrent comorbid risk factors [3–5]. Education about lifestyle behavior change is the priority if the patient's blood pressure is between 140/90 mm Hg and 160/100 mm Hg. Further, if the patient's blood pressure is ≥160/100 mm Hg, lifestyle management in conjunction with pharmaceutical interventions constitutes best practice [2].

Studies that investigate the outcomes of the interaction of the two research streams, that is, pharmacological and behavioral, are lacking. Given the unequivocal relationships among health behaviors and blood pressure, interactions between health behavior and drug efficacy are conceivable. Pharmacokinetics of hypertension drugs have been reported to be impacted by lifestyle factors [6]. If confounding lifestyle variables were controlled experimentally or their effects partitioned in post hoc analysis, drug effectiveness would be better elucidated. Biomedicine focuses on reducing the signs and symptoms of hypertension rather than the underlying causes and contributing factors. Furthermore, there is little evidence that drug therapy is simply additive to lifestyle modification; rather lifestyle modification can reduce reliance on medication [7].

The purpose of this systematic review was to establish how lifestyle modification is integrated into leading established antihypertension drug trials. Of interest were factors including smoking and stress reduction whose links to hypertension have been less well established compared with body weight and inactivity. Based on first-line clinical practice guidelines, the inclusion of lifestyle modification can not only be justified but also argued to be imperative, if related research is to be practice-informed. Specifically, we investigated to what extent and how lifestyle behaviors are systematically integrated into hypertension research paradigms of established hypertension drug trials and the extent to which lifestyle modification is masked by the use of nonspecific terms such as usual care. We contend that attention to lifestyle in the research paradigm of pharmacological studies related to common lifestyle-related conditions such as hypertension would enhance the practical implications and outcomes of such studies in terms of knowledge translation and outcome effectiveness versus simply efficacy and align them with real-world best practice recommendations.

## 2. Methods

### 2.1. Data Sources

The Cochrane Collaboration is an internationally recognized benchmark for high quality information based on best available evidence. Cochrane enables healthcare providers, policy makers, patients, and their advocates to make well-informed decisions about health care [28–31]. Therefore, we conducted an analysis of a current Cochrane Collaboration review informing first-line hypertension drug treatment. We searched the Cochrane Hypertension Group, a subgroup within the Cochrane library, and identified “First-line Drugs for Hypertension” [8] as the best representative article of a current systematic review investigating first-line drug care for hypertension.

### 2.2. Data Extraction and Synthesis

For each large clinical drug trial included in this review, Wright and Musini [8] identified a single major publication often the publication that included the final results for each trial. They identified 24 major publications. On preliminary review of each of these major publications, not all included a description of the methods of their research designs or referenced secondary source articles including the published methodology. To ensure that our analysis included all published information regarding the methodological design of the large clinical drug trials, secondary source articles from the drug trials were accessed. These articles were reviewed separately. Based on this criterion, 34 source articles resulted.

Each source article was reviewed for “mention of lifestyle interventions” including smoking cessation, diet, weight loss, physical activity and exercise, stress feduction, and moderate alcohol consumption. These lifestyle intervention categories were selected based on the 2012 Canadian Hypertension Education Program recommendations that are reported to be evidence based [32]. “Mention of lifestyle interventions” was defined as the inclusion of terms that corresponded with the six modifiable lifestyle behaviors. These terms could have been included as interventions in the study design or simply discussed in the introduction or conclusion of the paper. Other lifestyle interventions or undefined lifestyle modifications were classified as “other.” The term “any mention” included articles mentioned explicitly excluding any lifestyle modifications from their methodologies.

## 3. Results

Within the selected Cochrane review [8], 24 major publications corresponded to 24 large clinical drug trials. Of these, seven articles mentioned lifestyle modification. When the secondary sources were included, however, 11 of the 24 large clinical drug trials mentioned lifestyle modification within their related publications.

Table 1 shows the lifestyle modifications that were mentioned in the source studies [9–27]. The most common ones, in either the major publication or its secondary publication, were optimizing diet, smoking cessation, weight loss, and exercise. One major publication included mention of potassium supplementation that we categorized as “other” and one secondary publication referred to unspecified “lifestyle changes.” No articles mentioned alcohol restriction or relaxation/stress management to address secondary factors contributing to high blood pressure.

Of the 11 articles that mentioned lifestyle modification (46%), description of the interventions, their specific details and how they were systematically implemented, was lacking (Tables 2(a) and 2(b)). Inconsistent assessment of lifestyle behaviors and prescription of lifestyle modifications for research participants or lifestyle modification left to the judgment of researchers or doctors were recurring themes. Two of the published drug trials, Syst-Eur [21] and USPHSHCSG [25], systematically excluded lifestyle modification from their research designs or from being obligatory for the participants.

Finally, we observed no consistent relationship, however, between the publication dates and whether or not the drug trials considered lifestyle modification in their methodology.

## 4. Discussion and Conclusions

To our knowledge, this is the first investigation of methodological considerations related to evidence-informed first-line lifestyle modification in large established hypertension drug trials. Several points of discussion emerged regarding uncontrolled lifestyle modification within the designs of these leading drug trials.

The majority of articles in a leading recent source Cochrane review did not mention lifestyle modification. Not only the low level of integration of such considerations into drug research design is noteworthy, but also there appears to be no universal research paradigm to ensure trials parallel best clinical practice guidelines.

Four of the eleven drug trials that mentioned lifestyle modification (36%) did so only within their secondary sources; however, this methodology was not detailed in the corresponding major publication. In publications from the OSLO drug trial [13], for example, mention of general advice for weight loss, smoking, and salt consumption for their participants was included within the secondary publication, but no further mention was made in its major publication. This was often the case when the large drug trials resulted in multiple publications including separate publications specifically on the study design and rationale, while the major publications were often focused on the final results of the drug trials. Given that the major publications are likely the most read, the exclusion of mention of lifestyle modification methods in the major publications may be indicative of the perceived value of publishing the lifestyle modification considerations of the study design.

The Cochrane review by Wright and Musini [8] that we selected as a representative review of established high quality drug trials for first-line management of hypertension includes research from as early as 1966 with its most recent article being published in 2008. Of the large drug trials in the Cochrane review, 76% (26 of 34) were at least 20 years old. Newer articles however were no more likely to include mention of lifestyle than older ones.

A common feature of the source studies was a lack of details of the lifestyle modification interventions. Descriptions within the methods sections were nonspecific, for example, “avoidance or reduction of obesity will be advised” [14, 15], “encouraged to give up smoking” [23], and “individual investigator's judgment to prescribe lifestyle changes” [21]. Giving the effectiveness of lifestyle modifications depends on how they are performed, and understanding how they were administered, tracked, and monitored is critically important.

The lack of consistency observed for prescriptions of lifestyle modification across trials presents further challenges to interpreting the consistency and degree of emphasis that was placed on lifestyle and the degree to which modifiable factors confounded the study outcomes (despite these being randomized clinical trials). For example, SHEP [14] provided “standardized” advice on diet, exercise, smoking, and obesity, whereas VA-II [26] provided only salt restriction and activity restriction for patients with congestive heart failure. VA-NHLBI [27] provided dietary advice only in the presence of lipid abnormalities and advised everyone about the risks of smoking and obesity. Not only did the source studies vary with respect to the apparent inclusion of lifestyle modification, but also, if included, their parameters varied widely. Adding to these discrepancies were two publications that mentioned lifestyle in their introductions or conclusions but did not follow up with any form of intervention. The USPHSHCSG [25] drug trial, for example, mentioned only in the conclusion that “such careful follow-up supplemented by other hygienic intervention, such as weight control, moderate salt restriction, smoking cessation, and a reasonable exercise program, may be appropriate management for many mild, uncomplicated hypertensives, and avoids the potential hazards, inconvenience, and expense of long-term drug use." Although this insight was published in 1977, this knowledge appears not to have been translated and systematically integrated into established hypertension drug trials. By not controlling this established first-line intervention (i.e., lifestyle modification), it is not possible to establish the degree to which medications were superior, if at all, or the degree to which their effects may have been augmented. Whether participants could have avoided medications all together cannot be established.

Another factor that could affect the consistency of the lifestyle behavior change prescriptions was the way in which they were administered. Most source studies that provided lifestyle modification advice described it as either “general” or “standardized.” Due to the range of publication dates and the diversity of “standardized” protocols, these terms were ambiguous. Other studies however reported having had doctors or the investigators provide lifestyle modification advice on an individual basis. Given the powerful effects of lifestyle modification on normalizing blood pressure given its inclusion in best hypertension practice, researchers created potential confounding variables within the trials by not standardizing their lifestyle modification prescriptions. Although the MRC-TMH [10] drug trial recommended that doctors follow a consistent policy, the SYST-EUR [21] drug trial specifically discussed that diet and lifestyle changes should not be obligatory and did not define what was included under the “lifestyle changes” that were recommended by investigators. These inconsistencies and omissions, especially within the major publications, allow for misinterpretation of the findings given confounding lifestyle variables.

Only one drug trial, MRC-TMFH [11], reported performing a logistic regression analysis to adjust for risk factors such as smoking on drug outcomes. This analysis showed that the action of one of the antihypertensive medications assessed in the trial differed between smokers and nonsmokers. They reported that nonsmokers had a higher available amount of drug in their bodies. Wood [6], when discussing pharmacokinetic interaction of environmental factors with hypertension medication, used this example to support that nonsmokers may benefit more from certain hypertension medication than smokers [28]. As drug effects can be modified by lifestyle factors such as smoking, we contend that health indicators, such as nutrition and physical activity, could similarly influence drug outcomes, and thus warrant consideration when interpreting results.

One noteworthy source article that mentioned lifestyle factors was the UKPDS [23] drug trial, reported in 1991. This innovative study integrated a preliminary dietary regimen for blood glucose control for participants. Patients who were able to control their blood glucose through diet were excluded. This was the only trial to include lifestyle modification in its methodology and the only one to exclude patients based on their ability to follow a lifestyle modification regime. In this design, the investigators modeled what are currently the medical guidelines for the treatment of hypertension.

Of interest is the dearth of attention given to stress and its role in hypertension, in hypertension drug studies. Although stress is viewed as contributing to high blood pressure indirectly, autonomic activity is increased with activation of the sympathetically mediated fight/flight/fright mechanism, often the object of hypertensive medication. It could be argued that reducing this backdrop of elevated sympathetic activity might lower a person's threshold for clinical hypertension.

Several counterarguments could be posed given this analysis of RCCTs and the inclusion of lifestyle modification. One could argue that by design a RCCT homogenizes variation from known and unknown variables including lifestyle behaviors of drug trial participants thereby negating the need to consider lifestyle factors. Lifestyle modification has been long established as first-line intervention across all classifications of hypertension and concurrent comorbid risk factors, and thus, we believe drug trials need to shift to the next level of methodological sophistication and factor in lifestyle into their base designs. Given the power of lifestyle in preventing and reversing as well as managing hypertension [1, 2], pressing questions that need to be addressed relate to maximizing these benefits and eliminating the need for drugs, augmenting them with medication if indicated, that is, which medication for which patient in the presence of lifestyle behavior change (e.g., smoking cessation, nutrition, and physical activity), and understanding how healthy lifestyles impact the pharmacokinetics of the medications of interest.

A possible limitation of our analysis of the review by Wright and Musini [8] is that this Cochrane review is restricted to a subset of RCCTs, albeit it from a high quality established database; thus is not a comprehensive assessment of the methodology of all RCCTs investigating antihypertension drugs. Furthermore, although this review was published in 2009, some of the initial studies began as early as 1970. Study designs and ethics guidelines may have changed such that lifestyle modifications may need to be monitored and/or administered to all participants to analyze possible pharmacokinetics interactions. We noted with interest that one of the seminal studies that paid most attention to controlling lifestyle behaviors was published in 1977.

Although we have no reason to believe that comparable review of other trials would result in different findings, replication of other trials may have some benefit.

It is worth noting that, conversely, RCCTs examining the effects of lifestyle behavior change on blood pressure would be viewed as methodologically deficient if the confounding effects of medications were not controlled in some respect or built into the design of the trial.

Our findings have implications for the design of future studies. Foremost, we recommend that best practice hypertension guidelines need be reflected in hypertension studies; specifically, lifestyle modification strategies need to be a fundamental component of the research design of pharmacological studies on hypertension.

## Figures and Tables

**Table 1:** Characteristics of articles cited in a leading selected Cochrane review (Wright and Musini [8] that mentioned lifestyle modifications).

Lifestyle modification	Mentioned in major publication	Mentioned in secondary publication
Diet	6	5
Alcohol restriction	0	0
Exercise	4	2
Smoking cessation	4	3
Relaxation/stress Management	0	0
Weight loss	4	3
Other or undefined “lifestyle”	1	1

**Table:** (a)

Trial title	Publication date	Journal	Study design of publication	Lifestyle intervention mentioned (yes/no)	Diet	Alcohol restriction	Exercise
Carter	1970** [9]	The Lancet	Randomized to treatment or not	YES	“…treatment combined with restriction of salt intake…”	—	—
MRC-TMH	1992 [10]	British Medical Journal	Randomized single blind comparing 2 treatments and placebo (Final results)	NO	—	—	—
	1985** [11]	British Medical Journal	Randomized single blind comparing 2 treatments and placebo (Preliminary results)	YES	Doctors judgment for advising on salt intake	—	Doctors judgment for advising on exercise
OSLO	1986** [12]	Drugs	Open randomized to treatment or not	NO	—	—	—
	1980 [13]	American Journal of Medicine	Open randomized to treatment or not	YES	“General advice on salt consumption”	—	—
SHEP	1991** [14]	JAMA	Randomized, double blind, placebo controlled	NO	—	—	—
	1991 [15]	Hypertension: Supplemental Edition	Supplemental edition publication	YES	“Standardized general information on nutrition. Moderation of salt intake and emphasis on foods high in potassium are to be recommended”	—	Standardized general information regular gradual exercise
SHEP-P	1989** [16]	Stroke	Randomized double blind, placebo-controlled trial	NO	—	—	—
	1970 [17]	JAMA	Epidemiologic assessment of the role of blood pressure in stroke	NO	—	—	—
	1986 [18]	Controlled Clinical Trials	Recruitment experience review for SHEP-P publication	NO	—	—	—
	1982 [19]	Current Medical Research Opinion	Proposal/review of SHEP-P protocol publication	YES	Information on diet and exercise: “Moderation of salt intake to 3 to 4 g sodium daily in favour of foods high in potassium will be recommended”	—	Regular and graded exercise for muscular tone and skeletal mobility will be advised
SYST-EUR	1980** [20]	Lancet	Randomized, double blind, placebo controlled	NO	—	—	—
	1991 [21]	Aging	Objectives and protocol publication	YES	Individual investigators' judgment to prescribe diet changes	—	—
TEST	1995** [22]	Cerebrovascular Disease	Randomized, double blind, placebo controlled	YES	—	—	—
UKPDS 39	1991 [23]	Diabetologia	Study design publication	YES	“3 month trial of diet control after initial recruitment. All patients continued to receive dietary advice throughout the study and were encouraged to give up smoking…Centers were notified if the total cholesterol or triglyceride values were greater than 8.5 or 4.0 mmol/L respectively and could institute hypolipidemic therapy if dietary advice failed to lower these values satisfactorily” Initial diet therapy: “At the initial visit all patients were advised to take a “prudent” diet, containing approximately 50 % carbohydrate, low saturated fat and moderately high fiber with a reduced energy content if obese, aiming to attain ideal body weight. For the first 3 months they were seen at monthly intervals, usually by a dietitian as well as a doctor”	—	—
	1998** [24]	British Medical Journal	Randomized controlled (Open label)	YES		—	—
USPHSHCSG	1977** [25]	Circulation Research	Randomized, double blind, placebo controlled	YES	“There was no intervention on diet or smoking or other behavioral factors.” In conclusion, Moderate salt restriction may be appropriate	—	In conclusion, as a supplement, a reasonable exercise program may be an appropriate management
VA-II	1970** [26]	JAMA	Randomized, double blind, placebo controlled	YES	Low salt diet for congestive heart failure patients only	—	Restricted activity for congestive heart failure patients only
VA-NHLBI	1978** [27]	Circulation Research	Randomized, double blind, placebo controlled	YES	“Subjects were also advised if lipid abnormalities were present and a diet was recommended but not further emphasized”	—	At the discretion of the physician, nonhypertensive conditions warranting ambulatory therapy were treated or were referred to appropriate local facilities for evaluation and treatment

**indicates the major publication for the study as defined by Wright and Musini [8].

**Table:** (b)

Publication title	Smoking cessation	Relaxation/stress management	Weight loss	Other or undefined “lifestyle”	Description included in publication
Carter			“…treatment combined with …weight reduction.”		
MRC-TMH	Doctors' judgment for advising on smoking cessation		Doctors' judgment for managing obesity		
OSLO	“General advice regarding smoking”		“General advice regarding weight loss”		
SHEP	Standardized general information on smoking		Avoidance or reduction of obesity was to be advised	“Potassium supplements were given to all participants who had serum potassium concentrations below 3.5 mmol/L at two consecutive visits”	
SHEP-P			Avoidance or reduction of obesity will be advised		(On the third clinical visit)…A complete orientation to the trial is given, including information on diet and exercise… “Moderation of salt intake to 3 to 4 g sodium daily in favour of foods high in potassium will be recommended”
SYST-EUR				Individual investigators' judgment to prescribe lifestyle changes	“Diet and lifestyle changes have been recommended for the treatment of hypertension in the elderly, as they might be sufficient to reduce the blood pressure. However, few studies have specifically described the effects of such changes on blood pressure in the elderly and often no controls were included. In addition, aging may alter the relationships between the incidence of cardiovascular complications and indicators of cardiovascular risk, such as body weight and serum cholesterol” “…diet and lifestyle changes should not be obligatorily recommended to all patients. Individual investigators conserve the decision to prescribe diet and lifestyle measures to their patients in keeping with local treatment policies, provided that such measures are equally reinforced in all patients at a particular centre, independent of the level of blood pressure”
TEST	Only mentioned in introduction				
UKPDS 39	“Encouraged to give up smoking”				“3 month trial of diet control after initial recruitment. All patients continued to receive dietary advice throughout the study and were encouraged to give up smoking…Centers were notified if the total cholesterol or triglyceride values were greater than 8.5 or 4.0 mmol/L respectively and could institute hypolipidemic therapy if dietary advice failed to lower these values satisfactorily.” Initial diet therapy: “At the initial visit all patients were advised to take a “prudent” diet, containing approximately 50 % carbohydrate, low saturated fat and moderately high fibre with a reduced energy content if obese, aiming to attain ideal body weight. For the first 3 months they were seen at monthly intervals, usually by a dietitian as well as a doctor” “Type 2 diabetic patients aged 25–65 years inclusive, median age 53 years, median body mass index 28 kg/m^2^and median fasting plasma glucose 11.3 mmol/L, were recruited and treated initially by diet. Ninety five percent remained hyperglycaemic (fasting plasma glucose > 6 mmol/L) and were randomly allocated to different therapies”
USPHSHCSG	In conclusion, as a supplement, smoking cessation may be an appropriate management		In conclusion, as a supplement, weight control may be an appropriate management		Recommendations in conclusion: “Such careful follow-up supplemented by other hygienic intervention, such as weight control, moderate salt restriction, smoking cessation, and a reasonable exercise program, may be appropriate management for many mild, uncomplicated hypertensives, and avoids the potential hazards, inconvenience, and expense of long-term drug use”
VA-II					Recommendations in conclusion: “Such careful follow-up supplemented by other hygienic intervention, such as weight control, moderate salt restriction, smoking cessation, and a reasonable exercise program, may be appropriate management for many mild, uncomplicated hypertensives, and avoids the potential hazards, inconvenience, and expense of long-term drug use”
VA-NHLBI	All subjects were advised of the risks of smoking and obesity and, where indicated, were encouraged to eliminate these additional risk factors. Their responses were noted, but no further action was taken		All subjects were advised of the risks of smoking and obesity and, where indicated, were encouraged to eliminate these additional risk factors. Their responses were noted, but no further action was taken		

## References

[1] Musini V Evidence for practice: bringing best hypertension evidence to front line: first line treatments and second line treatments. http://hypertension.cochrane.org/evidence-practice.

[2] Daskalopoulou SS, Khan NA, Quinn RR (2012). The 2012 Canadian hypertension education program recommendations for the management of hypertension: blood pressure measurement, diagnosis, assessment of risk, and therapy. *Canadian Journal of Cardiology*.

[3] Bui Q (2010). First-line treatment for hypertension. *American Family Physician*.

[4] Chobanian AV, Bakris GL, Black HR (2003). Seventh report of the Joint National Committee on Prevention, Detection, Evaluation, and Treatment of High Blood Pressure. *Hypertension*.

[5] Whelton PK, He J, Appel LJ (2002). Primary prevention of hypertension: clinical and public health advisory from the National High Blood Pressure Education Program. *Journal of the American Medical Association*.

[6] Wood AJJ (1988). Drug interactions in hypertension. *Hypertension*.

[7] Appel LJ, Cooper LS, Obarzanek E (2003). Effects of comprehensive lifestyle modification on blood pressure control: main results of the PREMIER clinical trial. *Journal of the American Medical Association*.

[8] Wright JM, Musini VM (2009). First-line drugs for hypertension. *Cochrane Database of Systematic Reviews*.

[9] Carter AB (1970). Hypotensive therapy in stroke survivors. *The Lancet*.

[10] (1992). Medical Research Council trial of treatment of hypertension in older adults: principal results. MRC Working Party. *British Medical Journal*.

[11] (1985). MRC trial of treatment of mild hypertension: principal results. Medical Research Council Working Party. *British Medical Journal*.

[12] Leren P, Helgeland A (1986). Oslo hypertension study. *Drugs*.

[13] Helgeland A (1980). Treatment of mild hypertension: a five year controlled drug trial. The Oslo study. *American Journal of Medicine*.

[14] Probstfield JL (1991). Prevention of stroke by antihypertensive drug treatment in older persons with isolated systolic hypertension: final results of the Systolic Hypertension in the Elderly Program (SHEP). *Journal of the American Medical Association*.

[15] Borhani NO, Applegate WB, Cutler JA (1991). Part 1: rationale and design. *Hypertension*.

[16] Perry HM, McFate Smith W, McDonald RH (1989). Morbidity and mortality in the Systolic Hypertension in the Elderly Program (SHEP) pilot study. *Stroke*.

[17] Kannel WB, Wolf PA, Verter J, McNamara PM (1970). Epidemiologic assessment of the role of blood pressure in stroke. The Framingham study. *Journal of the American Medical Association*.

[18] Vogt TM, Ireland CC, Black D (1986). Recruitment of elderly volunteers for a multicenter clinical trial: the SHEP pilot study. *Controlled Clinical Trials*.

[19] McFate Smith W (1982). Isolated systolic hypertension in the elderly. *Current Medical Research and Opinion*.

[20] Reader R, Bauer GE, Doyle AE (1980). The Australian therapeutic trial in mild hypertension. Report by the management committee. *The Lancet*.

[21] Amery A, Birkenhager W, Bulpitt CJ (1991). Syst-Eur. A multicentre trial on the treatment of isolated systolic hypertension in the elderly: objectives, protocol, and organization. *Aging*.

[22] Eriksson S, Olofsson B, Wester P (1995). Atenolol for secondary prevention after stroke. *Cerebrovascular Diseases*.

[23] (1991). UK Prospective Diabetes Study (UKPDS). VIII. Study design, progress and performance. *Diabetologia*.

[24] (1998). Efficacy of atenolol and captopril in reducing risk of macrovascular and microvascular complications in type 2 diabetes: UKPDS 39. UK Prospective Diabetes Study Group. *British Medical Journal*.

[25] Smith WM (1977). Treatment of mild hypertension: results of a ten-year intervention trial. *Circulation Research*.

[26] Veterans Administration Cooperative Study Group on Antihypertensive Agents (1970). Effects of treatment on morbidity in hypertension. II: results in patients with diastolic blood pressure averaging 90 through 114 mm Hg. *Journal of the American Medical Association*.

[27] (1978). Evaluation of drug treatment in mild hypertension: VA-NHLBI feasibility trial: plan and preliminary results of a two-year feasibility trial for a multicenter intervention study to evaluate the benefits versus the disadvantages of treating mild hypertension: prepared for the Veterans Administration-National Heart, Lung, and Blood Institute Study Group for evaluating treatment in mild hypertension. *Annals of the New York Academy of Sciences*.

[28] The Cochrane Collaboration http://www.cochrane.org.

[29] Aertgeerts B, Cools F (2007). The Cochrane Collaboration and systematic literature reviews about the efficiency of a treatment. *Verhandelingen*.

[30] Moseley AM, Elkins MR, Herbert RD, Maher CG, Sherrington C (2009). Cochrane reviews used more rigorous methods than non-Cochrane reviews: survey of systematic reviews in physiotherapy. *Journal of Clinical Epidemiology*.

[31] Jørgensen AW, Hilden J, Gøtzsche PC (2006). Cochrane reviews compared with industry supported meta-analyses and other meta-analyses of the same drugs: systematic review. *British Medical Journal*.

[32] Hypertension Canada 2012 CHEP recommendations for management of hypertension. http://www.hypertension.ca/images/2012_CHEPFullRecommendations_EN_HCP1009.pdf.

